# Topical Oxaliplatin Produces Gain- and Loss-of-Function in Multiple Classes of Sensory Afferents

**DOI:** 10.1016/j.jpain.2023.07.023

**Published:** 2024-01

**Authors:** Nurjahan Saleque, Nisha Vastani, Clive Gentry, David A. Andersson, Mathilde R. Israel, Stuart Bevan

**Affiliations:** King's College London, Wolfson CARD, Institute of Psychiatry, Psychology & Neuroscience, London, UK

**Keywords:** Pain, paresthesia, chemotherapy-induced neuropathy, skin-saphenous nerve, ion channels

## Abstract

The platinum chemotherapeutic oxaliplatin produces dose-limiting pain, dysesthesia, and cold hypersensitivity in most patients immediately after infusion. An improved understanding of the mechanisms underlying these symptoms is urgently required to facilitate the development of symptomatic or preventative therapies. In this study, we have used skin-saphenous nerve recordings in vitro and behavioral experiments in mice to characterize the direct effects of oxaliplatin on different types of sensory afferent fibers. Our results confirmed that mice injected with oxaliplatin rapidly develop mechanical and cold hypersensitivities. We further noted profound changes to A fiber activity after the application of oxaliplatin to the receptive fields in the skin. Most oxaliplatin-treated Aδ- and rapidly adapting Aβ-units lost mechanical sensitivity, but units that retained responsiveness additionally displayed a novel, aberrant cold sensitivity. Slowly adapting Aβ-units did not display mechanical tachyphylaxis, and a subset of these fibers was sensitized to mechanical and cold stimulation after oxaliplatin treatment. C fiber afferents were less affected by acute applications of oxaliplatin, but a subset gained cold sensitivity. Taken together, our findings suggest that direct effects on peripheral A fibers play a dominant role in the development of acute oxaliplatin-induced cold hypersensitivity, numbness, and dysesthesia.

****Perspective**:**

The chemotherapeutic drug oxaliplatin rapidly gives rise to dose-limiting cold pain and dysesthesia. Here, we have used behavioral and electrophysiological studies of mice to characterize the responsible neurons. We show that oxaliplatin directly confers aberrant cold responsiveness to subsets of A-fibers while silencing other fibers of the same type.

## Highlights

• Oxaliplatin produces cold pain and hypersensitivity acutely.
• Many sensory nerve fibers treated with oxaliplatin lose touch sensitivity.
• A-fibers that retain touch sensitivity also display an abnormal cold sensitivity.
• Acute pain and discomfort may be a direct effect of oxaliplatin on sensory nerves.

Oxaliplatin is an effective anticancer drug for the treatment of colorectal cancer and other solid tumors.^1^, ^2^, ^3^, ^4^ Unfortunately, oxaliplatin produces characteristic cold-evoked paresthesia, dysesthesia and pain, which are reported as the key dose-limiting effect in approximately 85% of treated patients.^5^, ^6^, ^7^, ^8^, ^9^ These side effects are dose-dependent and regularly develop during or immediately after a single treatment.^10^, ^9^

Numerous ion channels (such as voltage-gated sodium, potassium, transient receptor potential channels [TRPs], and 2-pore domain potassium [K_2_P] leak channels) have been proposed to mediate oxaliplatin-induced cold hypersensitivity and paresthesias, based on investigations in vivo, and electrophysiological and molecular biology studies in vitro.^11^, ^12^, ^13^, ^14^ However, there is no consensus supporting any single target or ionic mechanism. Oxaliplatin has been shown to preferentially modulate the activity of A fibers,^14^, ^15^, ^16^, ^17^ and oxaliplatin treatment confers cold responsiveness to normally temperature-insensitive neurons.^18^ A similar population of neurons is recruited by other pharmacological agents*,* such as ciguatoxins, to cause cold-evoked pain behaviors in vivo, suggesting that ciguatoxins and oxaliplatin may engage common mechanisms.^18^, ^19^, ^20^ While it is clear that the acute actions of oxaliplatin are unlikely to be limited to a single class of sensory afferents, the sensory fiber types responsible for the abnormal cold responsiveness experienced by patients, and that observed in isolated sensory neurons, remain unknown. Here, we have explored the effects of oxaliplatin on peripheral sensory nerve terminals using skin-saphenous nerve recordings ex vivo and behavioral sensory testing in mice in vivo.

Our results show that mice treated with oxaliplatin display sensory hypersensitivities similar to those reported by patients. We further report that the application of oxaliplatin to the nerve terminals in the skin causes a distinct, complex pattern of functional abnormalities, particularly affecting A fibers. The activity of all A fiber subtypes tested, including low threshold mechanoreceptors, was altered by oxaliplatin treatment. A fibers treated with oxaliplatin either lost sensitivity (become refractory to mechanical stimulation) or fired more action potentials in response to mechanical stimulation. A fibers that remained mechanically responsive after oxaliplatin treatment additionally gained a novel, aberrant response to cooling. While proportions and response sizes differed, each A fiber class had at least one de novo cold-sensing fiber after oxaliplatin treatment. C fibers, which are central for normal cold sensing, were modestly affected by oxaliplatin treatment when applied directly to the receptive field. Similarly, skin-nerve preparations taken from animals treated with oxaliplatin suggested that multiple fiber types are affected. Taken together, these findings support the hypothesis that direct activation and silencing of peripheral sensory neurons by the platinum chemotherapeutic oxaliplatin underlie the acute hypersensitivities and dysesthesias experienced by patients.

## Methods

### Animal Behavior

Behavioral experiments were carried out according to the UK Home Office Animal Procedures (1986) Act. All procedures were approved by the King’s College London Animal Welfare and Ethical Review Body. Female C57BL/6J mice were obtained from Charles River (Margate, UK) or Envigo (Bicester, UK). Oxaliplatin (product Y0000271, Sigma-Aldrich) was dissolved in water containing 5% glucose (w/v) to recapitulate the iso-osmotic Cl^−^ free solution administered therapeutically. Mice were dosed with a single intraperitoneal (ip) injection of oxaliplatin (6 mg/kg) or vehicle (5% glucose). The effect of oxaliplatin on nociception was monitored for up to 7 days after administration, and mice were thereafter killed by cervical dislocation. The Randall-Selitto paw pressure test was performed using an Analgesy-meter (Ugo Basile, Gemonio, Italy). Mice were kept in their holding cages to acclimatize (10–15 minutes) to the experimental room. Mice were lightly restrained by the experimenter, and a constantly increasing pressure was applied to the dorsal surface of the hind paw using a blunt conical probe. The nociceptive threshold was defined as the force in grams at which the mouse withdrew its paw. A force cutoff value of 150 g was used to avoid tissue injury.

Cold nociception was assessed using a cold plate set to 10 °C to measure the paw withdrawal latency. Mice were lightly restrained, and one of the hind paws was placed in contact with the surface of the cold plate.^21^ The time taken for a withdrawal response was recorded. A latency cutoff value of 30 seconds was used to avoid tissue injury. Behavioral assessments were also performed to compare the sensitivities to different temperatures between vehicle- and oxaliplatin-treated mice on day 3 after injection. Paw withdrawal latencies were compared at 10 to 20 °C and 35 to 55 °C in 5 °C increments.

### Skin-Saphenous Nerve Recordings

The saphenous nerve and the dorsal hind paw skin that it innervates were dissected free to the lumbosacral plexus to ensure that a sufficient length of the nerve was available for recording. After dissection, the preparation was placed “inside up” or “outside down” in a bath chamber to facilitate oxygenation through the corium side of the skin, and the preparation was superfused with an oxygen saturated modified synthetic interstitial fluid (SIF) solution containing (in mM): 108 NaCl, 3.5 KCl, .7 MgSO_4_·7H_2_O, 26.2 Na_2_CO_3_, 1.65 NaH_2_PO_4_·2H_2_O, 9.6 sodium gluconate, 5.55 glucose, 7.6 sucrose, and 1.53 CaCl_2_ at 31 ± 1 °C. The preparation was left to equilibrate in the organ bath for an hour in order to limit spontaneous fiber activity. The skin was pinned down using insect pins before the start of recordings, and the saphenous nerve was pulled through into the adjacent smaller recording chamber and placed on a mirror. The recording chamber, including the mirror, and gold recording electrode with the saphenous nerve inside, was covered with paraffin oil to enable electrical isolation. The nerve was desheathed and subsequently teased into fine filaments with forceps. During the recordings, nerve filaments were placed on the gold electrode in the oil phase, and the reference electrode was positioned nearby but in the aqueous phase. Single fibers were identified with a mechanical search stimulus (manual probing to the skin using a blunt glass rod) and were recorded extracellularly using a low-noise differential amplifier. The conduction velocity of each axon was determined by electrically stimulating the most sensitive area of the receptive field with square-wave pulses (1 millisecond duration every 2 or 4 seconds for A and C fibers, respectively) using a fine electrode and a Digitimer stimulator (Digitimer, Welwyn Garden City, UK) and measuring the distance from receptive field to the recording electrode. In accordance with earlier studies and recordings in the mouse, a cutoff of 1.2 m/s was used to distinguish between myelinated and unmyelinated fibers.^22^ Fibers with a conduction velocity greater than 10 m/s were classified as Aβ fibers and those with a conduction velocity greater than 1.2 m/s and less than 10 m/s were classified as Aδ fibers. Fibers were further categorized into subclasses of populations based on responses to mechanical and cold stimulation following calculation of conduction velocity.

A mechanical stimulating probe (1 mm diameter) connected to a force transducer controlled by the Spike2 program (Cambridge Electronic Design [CED], Cambridge, UK) was placed in the same position as the electrode used for electrical stimulation. Forces (in g) were applied to the receptive field following conversion from an input value (in mV). The mechanical threshold was determined for each fiber by applying 2 seconds step waveform pulses. Forces were applied iteratively until the threshold, defined as the minimum force that evoked 2 action potentials was determined. Following this, fibers were mechanically stimulated with step protocols to enable a more detailed characterization of single units based on their adaptation properties. A 10 second step-shaped force was applied at a low force (4 g) and high force (15 g) with a 2 minute interval between applications to enable recovery of the fiber and to prevent tachyphylaxis. Step protocols were particularly important in distinguishing between low- and high-threshold mechanoreceptors (LTMR and HTMR respectively). After the mechanical characterization, the cold sensitivity of all fibers was assessed by isolating the receptive field of individual units with a metal ring (6 mm diameter). Vaseline was applied to the underside of the ring to create a tight seal and prevent leakages. The bath solution within the ring was manually removed with a syringe and a thermocouple was inserted into the ring to record temperature changes. Cold ramps (60 seconds) were applied by delivery of precooled SIF via a separate circulation system. If a fiber displayed spontaneous activity before the application of vehicle or oxaliplatin, it was excluded from further analysis. Oxaliplatin was made up on the day of the experiment from lyophilized powder (Sigma-Aldrich, St Louis, MI, USA) and dissolved in a 5% glucose solution (278 milliosmole). Control experiments (vehicle) used a 5% glucose solution. Oxaliplatin was used at a concentration of 600 µM based on work in rat skin-saphenous nerve preparations.^23^

The extracellular potentials were recorded using a World Precision Instruments low-noise head stage DAM80 AC differential amplifier (gain of 10^4^). The signal was filtered with a low pass (300 Hz) and a high pass (10 KHz) filter. The differential amplifier was connected to a CED 1401 acquisition board (CED). The output of the signal was visualized on an oscilloscope, recorded on a Personal computer, and made audible via speakers. All data were acquired using the Spike2 (CED) software.

### Data Analysis

Action potentials were analyzed in Spike2 by creating a template using the action potential waveform for the fiber in each recording. A separate channel was created with action potentials for these waveforms at each event (stimulation period) during the recording. This was duplicated and the channel draw mode function was used to convert this into action potentials/second (Hz) to visualize discharge rates as a histogram. For analysis, the period during mechanical and cold stimuli was scanned for events. Data were exported in 1 second time bins and basic analysis was performed to assess the total number of spikes, peak firing frequency and threshold (temperature for cold) of activation for cold and mechanical stimulations in Spike2, Excel (Microsoft, Redmond, WA, USA) and GraphPad Prism (version 9, GraphPad Software, Boston, MA, USA). In addition, the firing pattern of fibers during the cold and mechanical stimulation periods was exported. Data are presented as mean ± Standard error of the mean unless otherwise stated. Normality testing was performed on datasets and parametric or nonparametric hypothesis testing was conducted accordingly.

## Results

### Oxaliplatin Causes Cold and Mechanical Hypersensitivity In Vivo

**Figure 1:**
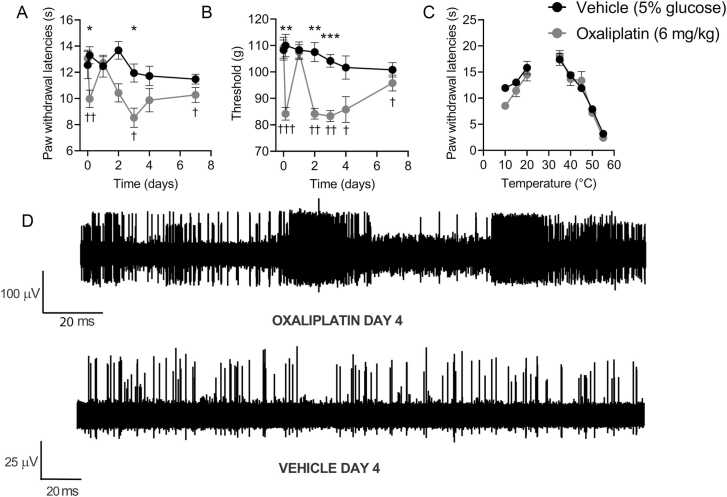
Systemic oxaliplatin causes biphasic mechanical and cold hypersensitivity in mice. Oxaliplatin (6 mg/kg in 5% glucose, ip) reduced the paw withdrawal latency in a 10 °C cold plate test (**A**) and the paw-pressure withdrawal (Randall-Selitto) threshold (**B**) 3 hours post administration compared to vehicle control. One day post administration, both cold and paw pressure sensitivities returned to baseline and control levels. Day 3 to 6 post injection, both 10 °C withdrawal latencies and paw-pressure thresholds were significantly less than the control (n = 6). (**C**) Oxaliplatin animals have reduced withdrawal latency compared to vehicle control animals in response to cold stimulation at 10 °C (*P* = .06) but not to warm or hot temperatures (day 3, n = 6). (**D**) Thinly teased filaments of the saphenous nerve from skin-nerve preparations taken from oxaliplatin-treated animals (day 3) displayed elevated levels of spontaneous activity. Representative trace from oxaliplatin-treated preparation illustrating nonstimulus evoked activity in putative A and C fibers including repetitive action potential volleys, which were not observed in preparations from vehicle treated mice. Data in **A** to **C** are mean ± SEM. *P < .05, **P < .01, ***P <.001, compared to vehicle, 2-way repeated measures ANOVA followed by Sidak’s correction. ^†^*P* < .05, ^††^*P* < .01, ^†††^*P* <.001, compared to the naïve preinjection value, 2-way repeated measures ANOVA followed by Dunnett’s test. Abbreviations: ANOVA, analysis-of-variance; SEM, Standard error of the mean.

Administration of oxaliplatin (6 mg/kg, ip) to mice produced cold and mechanical hypersensitivities (Fig. 1A and 1B).^11^ Mice treated with oxaliplatin displayed significantly reduced paw withdrawal latencies in a cold plate assay (10 °C) 3 hours after injection compared to the preinjection value (day 0) and compared to vehicle-treated mice (Fig 1A). In addition, the withdrawal threshold in the paw-pressure test was significantly decreased in oxaliplatin-treated mice 3 hours after injection, both compared to vehicle-treated mice and compared to the naïve baseline reading (day 0, Fig 1B). The behavioral sensitivities to noxious cold (10 °C) and mechanical stimulation followed a biphasic time course, with a period of marked remission following the immediate onset of hypersensitivities. One day after injection, oxaliplatin-treated animals were no longer hypersensitive to cold or paw pressure. This phase of brief remission was followed by sustained (2–7 days after treatment) hypersensitivities to both cold and mechanical stimulations (Fig. 1A and 1B). Oxaliplatin has been reported to produce hypersensitivity to heat, as well as mechanical and cold stimulations.^24^, ^6^ Therefore, we examined nocifensive behaviors over a range of innocuous and noxious temperatures 3 days after vehicle or oxaliplatin treatment (peak of oxaliplatin-induced hypersensitivity). Oxaliplatin did not alter the responsiveness to warmth or noxious heat in mice (35–55 °C) when compared to vehicle-treated mice (Fig 1C). The reduction in the paw withdrawal latencies between oxaliplatin-treated and vehicle-treated mice was significant at 10 °C, the coldest temperature tested.

### Oxaliplatin-Treated Skin Sensory Afferents are Hyperexcitable

We next turned to the skin-saphenous nerve preparation in an attempt to identify the sensory fiber types responsible for the mechanical and cold hypersensitivity observed in vivo. In preparations from mice treated systemically with oxaliplatin, single afferent units were difficult to isolate from finely teased nerve fibers due to a high level of spontaneous and evoked (both by mechanical probing and cooling of the receptive fields) activity in multiple A and C fiber afferents (Fig 1D). Oxaliplatin-induced hyperexcitability is thought to be predominantly mediated by A fibers.^14^ However, our preliminary observations of high levels of impulse activity in preparations from oxaliplatin-treated mice led us to hypothesize that both A and C fibers contribute to the oxaliplatin-evoked peripheral nerve hyperexcitability. The spontaneously active and multiunit nature of recordings from preparations obtained from mice treated systemically with oxaliplatin precluded investigations of specific identified afferent fiber classes (eg, Aδ, Aβ, down-hair [D-hair], etc). Since we were unable to accurately determine the conduction velocity of single units in preparations from oxaliplatin-treated mice, we could not characterize and classify these units reliably. Therefore, we adapted the experimental design to instead apply oxaliplatin directly to the receptive fields of sensory neurons in preparations from naïve mice after the identification and characterization of single units. In this way, we sought to define the acute, direct effects of oxaliplatin on specific, identified sensory nerve subtypes.

### Oxaliplatin Produced Profound A Fiber Abnormalities

We restricted our investigations of single fibers to mechanosensitive units since we used a mechanical search strategy to locate receptive fields. We have, therefore, not examined the potential impact of oxaliplatin on mechanoinsensitive CC fibers (C cold receptors). We have also not investigated responses evoked by heat since heat sensitivity is not a prominent complaint from patients and since we did not detect heat hypersensitivity in oxaliplatin-treated mice in vivo. In total, 96 A fibers were recorded and further categorized into 33 Aδ mechanoreceptors (AM, HTMRs), 16 Aδ—D-hair, 21 Aβ-slowly adapting fiber (AβSA), and 26 Aβ-rapidly adapting (AβRA) defined as previously described.^25^ Furthermore, we recorded 17 C mechanoreceptors (CM) and 20 C mechano-cold (CMC) receptors. These were divided between vehicle control and oxaliplatin (600 µM) treatment groups (control: n = 38 A fibers, 17 C fibers; oxaliplatin n = 58 A fibers, 20 C fibers).

**Figure 2:**
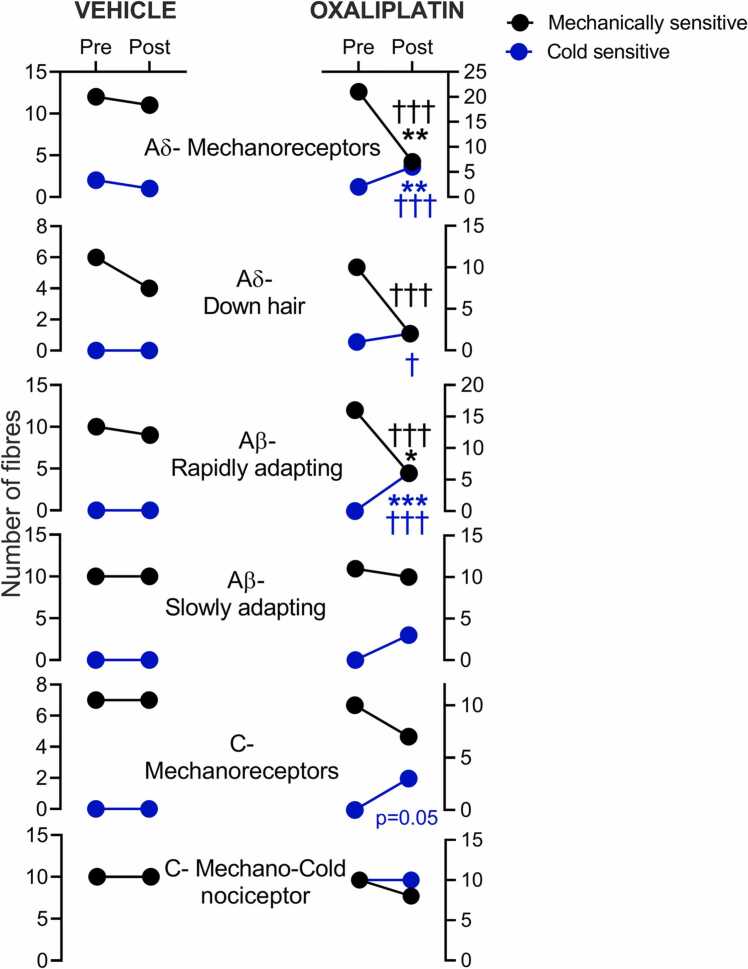
Oxaliplatin alters the proportions of cold and mechanically sensitive A fibers profoundly. The number of fibers that respond to mechanical (black circles) or cold (blue circles) stimulation after vehicle (left-hand panel) or oxaliplatin treatment (right-hand panel) of the corresponding receptive field. Before incubation with vehicle or oxaliplatin, all fibers were identified using mechanical stimuli and were thus initially mechanosensitive. After vehicle treatment (20 minutes, glucose 5% in SIF), almost all AM (11/12), D-hair (4/6), AβRA (9/10) AβSA (10/10), CM (7/7), and CMC (10/10) fibers fired in response to mechanical step stimulation. Pre or postvehicle treatment, very few A fibers fired action potentials in response to a 60 seconds cold ramp to ∼5 to 8 °C (pretreatment control 2/22 AM-C, post-treatment 1/11). CMC fibers responded to cold ramp before and after vehicle treatment (10/10), CM fibers did not gain de novo cold responsiveness after vehicle treatment (0/10). In contrast, significant proportions of AM, D-hair and AβRA were no longer sensitive to mechanical stimuli (*P* < .05, Fisher’s exact test) after oxaliplatin treatment (20 minutes, 600 µM in SIF). Remarkably, AM, D-hair and AβRA fibers that retained mechanical sensitivity almost all displayed a novel aberrant cold responsiveness. After oxaliplatin treatment, AβSA, CM, and CMC fibers retained mechanical sensitivity, but a subset of AβSA and CM fibers gained a novel cold sensitivity. *P < .05, **P < .01, ***P < .001, compared to vehicle, Fisher’s exact test. ^†^*P* < .05, ^††^*P* <.01, ^†††^*P* < .001, compared to pretreatment, Fisher’s exact test. Abbreviation: AβSA, Aβ slowly adapting fiber.

Vehicle treatment did not significantly change the sensitivity of A and C fibers to mechanical or cold stimulation when compared to pretreatment (Fig 2, left-hand panel). In contrast, oxaliplatin (600 µM) reduced the number of fibers that remained mechanically responsive after treatment (AM: oxaliplatin 7/21, vehicle 11/12, *P* < .01, Fisher’s exact test; D-hair: oxaliplatin 2/10, vehicle 4/6; AβRA: oxaliplatin 6/16, vehicle 9/10, *P* < .05, Fisher’s exact test). Crucially, fibers that belonged to these A fiber classes and retained mechanical responsiveness after oxaliplatin treatment almost uniformly gained a novel aberrant cold sensitivity (6/7 AM, 2/2 D-hair, 6/6 AβRA; Fig 2, right-hand panel, blue dots). AβSA fibers resisted oxaliplatin-induced silencing, and a more modest proportion became cold-sensitive after oxaliplatin treatment (3/10). The loss of mechanical CM fiber responsiveness following oxaliplatin was similar to that observed in vehicle-treated preparations, but we observed a novel aberrant cold sensitivity in 3/7 CM fibers after oxaliplatin treatment. The number of CMC fibers responding to mechanical and cold stimulation before treatment was 10 and 10, respectively, compared to 9 and 10 after oxaliplatin treatment. Likewise, vehicle-treated CMC fibers did not lose sensitivity to mechanical and cold stimulation, and 10/10 responded to mechanical and cold stimulation after treatment (Fig 2). We next examined the firing properties of the fiber classes available after oxaliplatin treatment.

### Adaptation Properties to Mechanical Stimulation of Some A Fibers are Profoundly Altered by Oxaliplatin

#### Aδ mechanoreceptor fibers

**Figure 3:**
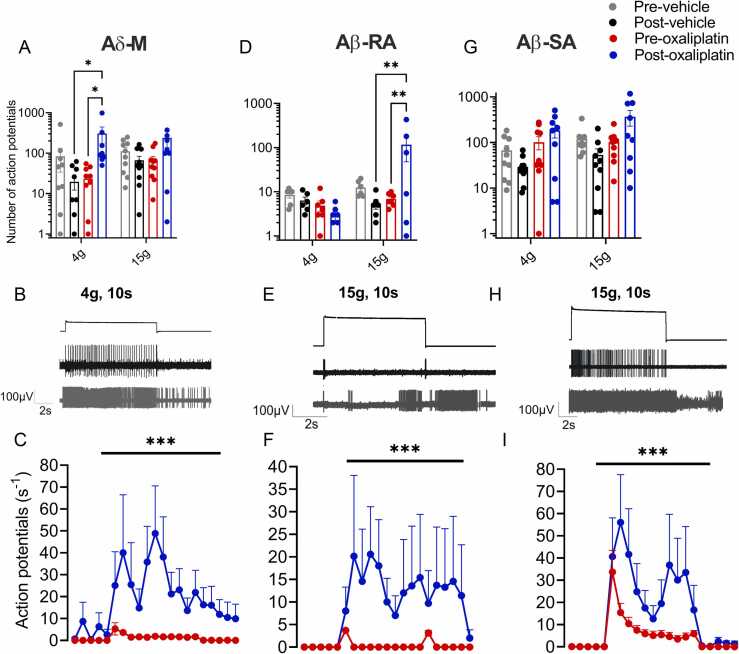
A-fibers that retained mechanical responsiveness after oxaliplatin exhibit increased firing rates and altered adaptation properties. The number and frequency of action potentials discharged during 10 seconds mechanical stimuli are increased post oxaliplatin (blue circles) treatment compared to pretreatment (red circles) baseline and post vehicle control values (black circles). (**A**) AM fibers that retained responsiveness after exposure to oxaliplatin (600 µM in SIF) fired significantly more action potentials during the 4 g step stimulus compared to vehicle-treated units and compared to pretreatment (*P < .05, Mixed-effects analysis followed by Tukey’s test, n = 8–10 fibers). (**B**) AM fibers treated with oxaliplatin displayed altered adaptation patterns, such as firing after removal of the 4 g stimulus (n = 5/7), which was not seen before treatment. (**C**) Average adaptation properties to 4 g, 10 seconds mechanical force step from all AM fibers tested (***P < .001, Wilcoxon’s test, n = 10). (**D**) AβRA fibers fired more action potentials during 15 g steps after oxaliplatin treatment compared to vehicle-treated units and compared to pretreatment (Mixed-effects analysis followed by Tukey’s test, n = 6–7 fibers). (**E**) Oxaliplatin changed the adaptation of AβRA fibers (observed in 3 of the 6 units), which typically do not fire throughout the mechanical stimulus (pretreatment and postvehicle control). Some oxaliplatin-treated AβRA fibers also fired after the removal of mechanical stimulus (2/6). (**F**) Average adaptation properties in response to 15 g, 10 seconds mechanical force step from all AβRA fibers tested (Wilcoxon’s signed rank test, n = 7). (**G**) AβSA fibers retained their adaptation properties but fired more action potentials after oxaliplatin in response to 15 g force steps compared to post vehicle and preoxaliplatin (Mixed-effects analysis followed by Tukey’s test, n = 10). (**H**) Representative trace of AβSA activity during a 15 g mechanical stimulus. (**I**) The average adaptation properties of AβSA fibers (n = 10) demonstrate an increased firing rate, particularly at the beginning and end of the mechanical stimulation (Wilcoxon’s signed rank test, n = 10). Abbreviation: AβSA, Aβ slowly adapting fiber.

Oxaliplatin significantly and substantially increased the number of action potentials in AM fibers elicited by a 4 g force step compared to vehicle and pretreatment controls (post vehicle control 20 ± 7; preoxaliplatin 23 ± 6; postoxaliplatin 308 ± 137 Action potentials (APs), *P* < .05, Kruskal-Wallis test, n = 10, Fig. 3A–3C). After exposure to oxaliplatin, the firing rate evoked by 15 g force was not significantly increased (oxaliplatin 239 ± 136, vehicle 66 ± 17, *P* > .05, Kruskal-Wallis test, n = 10). Responses to stimulation with 15 g were also no larger than those evoked by stimulation with 4 g, suggesting that the encoding properties of AM units had been compromised. Some AM fibers also displayed activity outlasting the stimulation period at both low and high force (4 g; 5/10, 15 g; 2/10, example given in Fig 3B). In contrast to the increased number of action potentials observed in response to each of the stimuli after oxaliplatin treatment, vehicle treated AM fibers fired fewer action potentials in response to all forces tested after vehicle treatment (4 g pretreatment 84 ± 50 vs posttreatment 20 ± 7 APs, n = 10, data not shown and Fig 3A; 15 g, pretreatment 111 ± 26 vs posttreatment 67 ± 17 APs; *P* > .05 Kruskal-Wallis followed by Dunn’s multiple comparison test, n = 10).

#### Down-hair fibers

A small number of D-hair low threshold afferents were recorded post oxaliplatin and vehicle treatment (data not shown, see Fig 2 for proportions). The number of D-hair units that were mechanically available after a 20-minute application of the vehicle was reduced by ∼30% (4/6). This reduction increased to 80% after oxaliplatin treatment (2/10 remained mechanically sensitive). The 2 units that remained mechanically sensitive post oxaliplatin did not fire more action potentials or change adaptation properties compared to vehicle-treated units.

#### Aβ-rapidly adapting fibers

Typically, murine AβRA fibers respond to mechanical force with an on-off pattern of impulse activity.^25^ However, oxaliplatin altered the adaptation properties to stimulation with higher force steps in a subset (3/7) of the RA fibers that retained mechanical responsiveness after treatment. In these fibers, the response to 15 g mechanical force was reminiscent of that of SA fibers, with sustained activity throughout the 10 seconds force step (Fig. 3D–3F). Oxaliplatin treatment of AβRA receptive fields also led to the impulse activity persisting beyond the withdrawal of the force transducer in some units (2/7, Fig 3E). Oxaliplatin did not increase the total number of action potentials generated during application of 15 g mechanical force compared to vehicle treatment (post vehicle control 5 ± 1; preoxaliplatin 7 ± 1; postoxaliplatin 116 ± 69 APs, n = 6–7 fibers, Fig 3D), however the responses were very heterogeneous (max-min, vehicle 11-2 APs; oxaliplatin 432-1 APs, Fig 3D, *P* > .05, Kruskal-Wallis test). Firing evoked by a 4g step did not increase after the corium was treated with vehicle or oxaliplatin (prevehicle control 9 ± 1 vs post vehicle control 6 ± 1 APs, data not shown and Fig 3D, n = 6; preoxaliplatin 5 ± 1 vs postoxaliplatin 3 ± 1 APs, Fig 3D, n = 6–7).

#### Aβ-slowly adapting

The impulse activity evoked by force steps in AβSA fibers was reduced significantly after vehicle treatment (4 g, 15 g; prevehicle control 67 ± 19, 120 ± 25, APs vs postvehicle control 29 ± 6, 53 ± 19, 45 ± 13 APs, n = 10, *P* < .01 for 15 g, Friedman’s followed by Dunn’s post hoc test, Fig 3G). In contrast, the impulse activity was maintained but not significantly increased in AβSA fibers after oxaliplatin treatment compared to pretreatment values (4 g, 15 g; preoxaliplatin treatment 102 ± 32, 102 ± 21, 139 ± 27 APs, n = 10 vs postoxaliplatin 184 ± 27, 330 ± 129, 389 ± 135 APs, n = 10, *P* > .05 Friedman’s followed by Dunn’s post hoc test, Fig 3G). The total numbers of action potentials discharged during 4 g and 15 g force steps were also not significantly increased after oxaliplatin compared to the vehicle (Fig 3G, *P* > .05, Kruskal-Wallis followed by Dunn’s post hoc test), which is likely due to the underlying heterogeneity of AβSA subclasses and responses.^26^, ^27^

AβSA fibers fired at higher rates at the beginning of the 10 seconds force step in control conditions.^28^, ^29^, ^30^ This adaptation property was maintained after oxaliplatin treatment; however, on average firing rates increased at the end of the force step (Fig 3H). Unlike other A fiber classes, none of the studied AβSA fibers fired after the removal of the mechanical stimulus (Fig. 3H and 3I).

### Oxaliplatin Does Not Alter C Fiber Activity Evoked by Mechanical Stimulation

**Figure 4:**
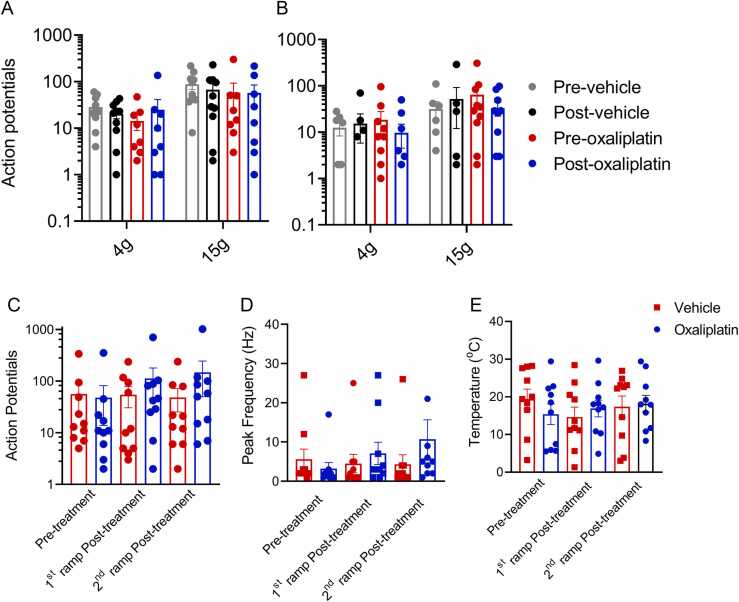
Oxaliplatin did not alter the firing rate or cold activation thresholds in C-fibers. Oxaliplatin treatment did not affect the firing rate evoked by mechanical force steps in CM or CMC units. CMC (**A**) and CM (**B**) fibers respond to mechanical force steps by firing similar numbers of action potentials before and after vehicle and oxaliplatin (*P* > .05, 2-way RM ANOVA, n = 7–10). The number of action potentials (**C**) and the peak firing frequency (**D**) evoked by cold in CMCs were unchanged by oxaliplatin or vehicle treatment (n = 10). (**E**) The temperature threshold for cold activation of CMCs was unchanged by vehicle or oxaliplatin treatment (n = 10). Abbreviation: ANOVA, analysis-of-variance.

Canonical cold and “pain” sensing afferents are C fibers.^31^, ^22^ Therefore, we examined the effect of oxaliplatin on the firing rate and pattern of CM and CMC fibers in response to cold and mechanical stimuli. Unlike A fiber afferents, exposure to oxaliplatin did not alter CMC and CM fiber responses to mechanical stimulation (Fig. 4A and 4B). CMC fibers were stable during control conditions (4 g, 15 g; prevehicle control 28 ± 6, 88 ± 20 vs postvehicle control 20 ± 4, 67 ± 22, APs, data not shown and Fig 4A, *P* > .05, Kruskal-Wallis followed by Dunn’s post hoc test) and the action potential firing rate did not change significantly after oxaliplatin treatment (4 g, 15 g, preoxaliplatin 14 ± 5, 58 ± 35 vs postoxaliplatin 25 ± 16, 59 ± 27, APs, Fig 4A, *P* > .05, Kruskal-Wallis followed by Dunn’s post hoc test). Similarly, CM fibers did not fire more action potentials during 4 g and 15 g steps after oxaliplatin treatment (4 g, 15 g; preoxaliplatin 19 ± 9, 64 ± 29, vs post oxaliplatin 10 ± 5, 33 ± 11, APs, n = 10, *P* > .05, Kruskal-Wallis followed by Dunn’s post hoc test, Fig 4B) or vehicle treatment (4 g, 15 g, prevehicle control 12 ± 4, 32 ± 14, APs vs postvehicle control 15 ± 9, 52 ± 30, Kruskal-Wallis followed by Dunn’s post hoc test, Fig 4B). CMC, and to a lesser extent, CM fibers were also largely resistant to oxaliplatin-induced mechanical silencing that was observed in several A fiber classes (see Fig 2).

### Oxaliplatin Confers Aberrant Cold Sensitivity to a Subset of Cold-insensitive CM Fibers

Application of oxaliplatin to the receptive fields of CMC fibers did not significantly affect the number of action potentials discharged during a subsequent cold ramp (preoxaliplatin treatment 48 ± 34, 1st cold ramp after oxaliplatin 113 ± 67, *P* > .05, Kruskal-Wallis followed by Dunn’s post hoc test, n = 10, Fig 4C). This number was not increased significantly in response to a 2nd cold ramp after oxaliplatin (147 ± 97, Fig 4C). The firing frequency in vehicle-treated CMC fibers remained stable throughout the recording period (Fig 4D). We next asked whether the temperature threshold for cold activation of these CMC fibers was altered by oxaliplatin treatment. The temperature threshold of activation was defined as the temperature at which the second of 2 sequential action potentials was elicited. The average thresholds, which ranged between ∼14 to 20 °C, were not significantly altered by treatment with either vehicle or oxaliplatin (prevehicle treatment 19.4 ± 2.6, 1st cold ramp post vehicle 14.6 ± 2.6 and 2nd cold ramp post vehicle 17.4 ± 2.8 °C vs preoxaliplatin treatment 15.4 ± 2.7, 1st cold ramp post vehicle 16.9 ± 2.2 and 2nd cold ramp post vehicle 18.2 ± 2.2 °C, *P* > .05, Kruskal-Wallis followed by Dunn’s post hoc test, n = 10, Fig 4E). Some CM fibers (3/7) responded to cold after oxaliplatin treatment compared to 0/10 after vehicle. The number of action potentials evoked by the cold ramp, as well as the peak firing frequency, was low compared to CMC levels (pretreatment 0, 1st cold ramp post vehicle 8 ± 1, 2nd cold ramp post vehicle 29 ± 9 APs, n = 2–3).

### Oxaliplatin Treatment Conferred Cold Sensitivity to Subsets of all A Fiber Subtypes

**Figure 5:**
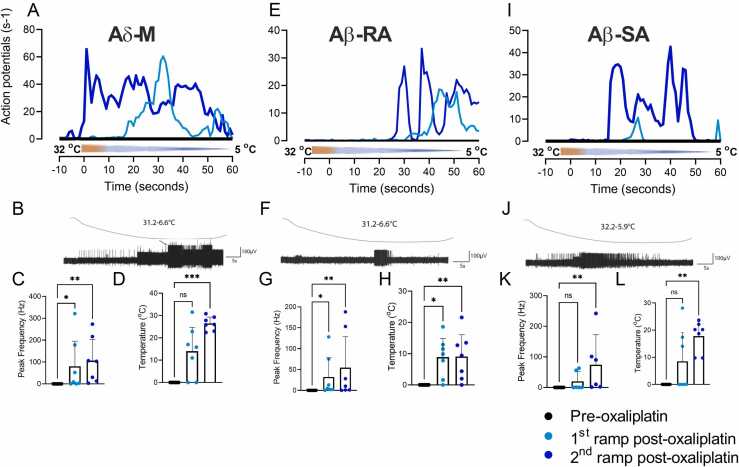
Aberrant cold sensitivity of oxaliplatin-treated AβSA, AβRA, and AM fibers. Application of oxaliplatin to receptive fields of A fibers conferred aberrant sensitivity to stimulation with a cooling ramp (from 32 °C to less than 10 °C, during 60 seconds). None of the A-fibers represented here responded to cold before oxaliplatin treatment. (**A**, **E**, **I**) Lines represent average firing frequencies for each A fiber type. Light blue lines are responses to the first cold ramp after oxaliplatin treatment, dark blue line represents responses to second cold ramp posttreatment. Typical responses evoked by cooling in AM (**B**), AβRA (**F**), and AβSA (**J**) fibers are shown together with the respective peak firing frequencies (**C**, **G**, **K**) and temperature response thresholds (**D, H, L**). The second cold ramp generated cold-evoked activity at warmer temperatures than the first ramp in AM and AβSA units (both *P* < .05, Wilcoxon matched-pair signed rank test, n = 7). Abbreviation: AβSA, Aβ slowly adapting fiber.

In the mouse skin, low threshold A beta fibers (AβRA and AβSA) are normally insensitive to cold or cooling stimuli, and only a small proportion of Aδ (AM and D-hair fibers) are considered cold-responsive.^22^ In contrast, oxaliplatin conferred cold responsiveness to a subset of units from all classes of A-fibers and to some previously cold-insensitive CM-fibers (see Fig 2). Oxaliplatin treatment conferred a novel, aberrant cold sensitivity to 16/23 previously cold-insensitive A fibers in response to cooling to less than 10 °C compared to 0/34 vehicle control fibers (AM; Fig. 5A–5D, AβRA; Fig. 5E–5H, AβSA; Fig. 5I–5L). Overall, these novel cold responses were mostly observed in A fibers that also retained mechanical sensitivity after oxaliplatin (11/16). However, of the cold-responsive oxaliplatin-treated A-fibers, 1 AM, 1 D-hair, 3AβRA were refractory to mechanical stimulation after the cold ramp. The magnitude of cold responses in oxaliplatin-treated A fiber receptive fields was highly variable (Fig 5). Some A fibers were unresponsive (fired ≤2 APs) to cold during the first ramp but fired during the second cooling period and so were considered cold sensitive and included in the following analysis. Repeated exposure to cold progressively enhanced responses in all types of A fibers after treatment with oxaliplatin, and the average response threshold was raised to warmer temperatures during the second ramp after oxaliplatin treatment. The peak firing frequencies for AM fibers were 1st cold ramp 80.4 ± 48.9 and 2nd cold ramp 106 ± 39.4 APs s^−1^; AβRA 1st cold ramp 32.1 ± 18.9 and 2nd cold ramp 54.4 ± 30.4 APs s^−1^; AβSA 1st cold ramp 20 ± 12.5 and 2nd cold ramp 74.3 ± 38.2 APs s^−1^ (Fig 5, n = 6–7). In control conditions, a small number of AM fibers are cold sensitive in the mouse (in a study by Zimmermann et al;^22^ this study, vehicle 2/13; oxaliplatin 2/22). These AM-C fibers were excluded from the analysis of de novo cold responses (Fig 5). The temperature thresholds of activation of de novo cold sensing AM fibers were 1st cold ramp after oxaliplatin 14.0 ± 4.3 and 2nd cold ramp 26.4 ± 1.1 °C; AβRA 1st cold ramp 8.9 ± 2.6 and 2nd cold ramp 9.1 ± 2.9 °C; AβSA 1st cold ramp 8.5 ± 4.7 and 2nd cold ramp 17.8 ± 2.3 °C (Fig 5, n = 6–7). Repeated exposure of A fiber receptive fields to cold did not lead to action potential firing after vehicle treatment (AM n = 13, DH n = 6, AβSA n = 10, AβRA n = 10).

## Conclusions

Oxaliplatin treatment produces unpleasant sensory abnormalities acutely, including hyperalgesia, paresthesia associated with numbness, cold-evoked pain, and dysesthesia. These acute sensory abnormalities remain dose-limiting side effects and a debilitating problem for patients. In this study, we show that application of oxaliplatin to the receptive fields of A, and to a lesser extent C fibers, confers an aberrant cold responsiveness. Oxaliplatin sensitized a subset of AM, AβSA, and AβRA to cold and mechanical stimuli, and we observed cold-evoked responses in previously cold-insensitive C fibers after treatment with oxaliplatin. Furthermore, oxaliplatin made a significant proportion of A fibers refractory to mechanical stimulation. We conclude that the pain, paresthesia, and numbness experienced by patients at the time of infusion may be caused by the dual activation and silencing of peripheral sensory neurons.

In mouse models of oxaliplatin-induced neuropathy and acute sensory abnormalities, intraplantar and ip dose regimens have both been shown to enhance behavioral responsiveness to cold and mechanical stimuli.^12^, ^32^, ^33^ We show that oxaliplatin (ip) gives rise to cold and mechanical hypersensitivities within 3 hours of administration, which parallels the rapid onset of sensory abnormalities observed clinically.^8^ Surprisingly, both of these behavioral parameters spontaneously returned to the preadministration level 24 hours after oxaliplatin administration. To our knowledge, this is the first time that apparent remission of cold and mechanical hyperalgesia has been reported in a mouse model of oxaliplatin-induced hyperalgesia. Experimental models of formalin-induced pain are characterized by a biphasic time course of the behavioral phenotype^34^, ^35^ albeit on a shorter scale. The first phase of the formalin test is thought to be caused by direct activation of peripheral sensory neurons. It is followed by a sustained second phase, thought to be driven by both central sensitization and altered peripheral input.^34^ Formaldehyde directly applied to rat afferents causes transient excitation of C and A fibers followed by loss of excitability, including a loss of mechanical sensitivity.^36^ In the same study, the C but not A fiber afferents recovered some activity post conduction block, ∼30 minutes after removal of formaldehyde. Higher activity of Aβ fibers was observed from whole sural nerve recordings during the first phase of formalin-induced pain compared with phase 2. Our results suggest that the sensory abnormalities produced by the administration of oxaliplatin may follow a similar course of induction, whereby the initial phase is caused by direct activation and sensitization of some peripheral nociceptors (as demonstrated here by direct application to receptive fields in the skin); followed by a sustained sensitization caused by altered peripheral input. Preparations taken from animals treated with oxaliplatin (3 days after injection) displayed high levels of spontaneous activity in both C and A fibers, which is consistent with this hypothesis. The spontaneous activity observed in preparations from oxaliplatin-treated mice also appears to be a likely source of hypersensitivity and paresthesia in vivo. The temporary remission in nocifensive behaviors observed in this study remains unexplained. While it is highly unlikely that formalin and oxaliplatin share mechanisms of action in vivo, the formalin model does provide an example of a 2-phase rodent pain model in which the phases are distinguishable in skin afferent recordings like those performed in this study.^36^

It is estimated that ∼90% of all patients treated with oxaliplatin report paresthesia, pain, and cold-evoked dysesthesias, often at the time of infusion.^8^ Central sensitization caused by oxaliplatin has been reported to contribute to mechanical, but not cold hypersensitivity in humans.^6^ However, prophylactic pregabalin failed to attenuate pain in patients receiving oxaliplatin infusions, suggesting that the early phase may not rely on central mechanisms.^37^ Since pain may last for years after cessation of oxaliplatin treatment, long-term changes in neuronal plasticity and function similar to those observed in neuropathies of other etiologies are likely to be involved. The changes to Aβ fiber properties reported here may explain the paresthesias and numbness experienced by patients after a single treatment cycle.^8^ Studies using Quantitative Sensory Testing have shown that long-term treatment of oxaliplatin causes deficits in Aβ fiber function by increasing detection thresholds in the bump test and to von Frey filaments.^15^ Patients experience mechanical and tactile allodynia, which might, in part, be explained by the sensitization of Aβ-LTMRs and AM fibers.^15^, ^38^ The loss of activity in medium-large diameter fibers observed in this study after relatively brief incubation with oxaliplatin might also contribute to numbness experienced by patients during chemotherapy infusions. Silencing of fibers shown in this study, 20 minutes after oxaliplatin administration, may appear incongruous with the high levels of activity seen in fibers from animals treated with oxaliplatin (day 4). However, some fiber types (Aβ-SA, C fibers) resisted silencing, and this subset could underlie the heightened activity at later time points. We also cannot rule out that silenced fibers recover function over time, thereby contributing to sensory hypersensitivity in vivo.

The major finding of this study is that oxaliplatin applied directly to sensory nerve terminals either silences or confers de novo cold responsiveness in Aβ-LTMRs and AM nociceptors. This adds to the literature supporting the critical role of A fibers in oxaliplatin-induced cold-evoked pain.^16^, ^14^ Why are A fibers particularly vulnerable to oxaliplatin treatment? Mechanisms such as direct modulation of ion channels (not least Na_V_1.6) and dysfunction in mitochondrial function have been proposed.^14^, ^39^ Large diameter A fibers express high levels of Na_V_1.6,^40^, ^41^ and selective inhibition of Na_V_1.6 reversed the acute oxaliplatin-induced cold and mechanical allodynia in mice.^12^ Oxaliplatin causes enhanced Na_V_1.6 resurgent current in large diameter dorsal root ganglia (DRG) neuron,^14^ and Na_V_1.6 selective toxins that cause enhanced resurgent currents in large diameter DRG lead to preferential activation of A fibers in skin-saphenous nerve recordings.^40^ Indeed, burst firing patterns akin to those seen in some oxaliplatin-treated A fibers were observed after selective Na_V_1.6 activation.^40^ Na_V_1.6 is also expressed in peptidergic and nonpeptidergic DRG neurons. However, Na_V_1.6 activation alone does not cause cold allodynia in vivo and conversely dampened temperature-evoked C fiber responses ex vivo.^12^, ^40^

In rodent models, repeated oxaliplatin treatment has been reported to increase expression of Transient receptor potential melastatin 8 and Transient receptor potential ankyrin 1, and reduce expression of the potassium channels TWIK-related K^+^ channel 1 and TWIK-related Arachidonic Acid-Stimulated K^+^.^42^, ^11^, ^43^, ^44^, ^45^, ^46^ Alterations of the expression or function of these ion channels would primarily be expected to affect the excitability of C fibers, which we did not observe in our study, almost certainly because we applied oxaliplatin locally to the skin and only for 20 minutes. This short duration and restricted site of application (peripheral terminal) contrast with the more sustained systemic dose regimens that demonstrated transcriptional regulation of ion channels in DRGs. In addition to exciting neurons by acting at specific transduction molecules (stimulating Transient receptor potential melastatin 8,^47^, ^48^ inhibiting Potassium channel subfamily K member (KCNKs)^49^), cooling also inhibits K_v_ channels,^50^, ^51^ thereby increasing the neuronal excitability by increasing the membrane input resistance.^50^, ^51^ In this way, blockade of K_v_1 channels reportedly unmasks previously silent large diameter cold sensing neurons in the DRG^18^ and causes cold allodynia in vivo.^12^ This effect is potentiated by a Na_V_1.6 activator that preferentially modulates the activity of large mechanosensitive DRG.^12^ Our findings are consistent with the hypothesis that enhanced cold and mechanical sensitivity produced by oxaliplatin in vivo is predominantly driven by large diameter A fibers, which express both Na_V_1.6 and multiple K_V_ channels.

Recently, in vivo calcium imaging of DRG neurons in mice treated with oxaliplatin and exposed to cold stimuli revealed “silent” cold-sensitive afferents at the level of the DRG.^18^ These de novo cold-sensitive neurons are predominantly Na_V_1.8-expressing medium to large diameter (greater than 400 µm^2^) however, only few expressed *Ntrk2* and *Calb*1, reported to be molecular markers for Aδ-fiber LTMRs (D-hair fibers) and Aβ-fiber LTMR respectively.^18^ In our study, oxaliplatin altered the properties of both AM, D-hair and Aβ LTMRs profoundly, either by conferring an aberrant cold sensitivity or silencing these units. Our observations thus suggest that in addition to the Na_V_1.8-expressing medium to large diameter fibers that gained cold sensitivity in vivo Ca^2+^-imaging experiments,^18^ other A-fibers were also affected by local applications of oxaliplatin. A key difference between this report and the study by MacDonald et al,^18^ is the time after oxaliplatin administration (20 minutes after application to receptive field, compared to 3 hours after intraplantar injection) when cold responsiveness was tested. Furthermore, low threshold mechanoreceptor classification in our study is performed after functional analysis of the afferent firing pattern. The relationship between transcriptional markers and afferent properties has not been extensively validated functionally and may confound the interpretation of the results. MacDonald et al^18^ report that the large diameter de novo cold sensing neurons also responded to pinch stimuli. Consistent with these observations, we find that A fibers that respond to cold after oxaliplatin treatment almost always retained mechanical responsiveness and responded to noxious (high force) mechanical stimuli. The scope of future work should include an analysis of the K_V_ conductances that may be responsible for oxaliplatin-induced activation and cold sensitivity of peripheral sensory neurons.

In conclusion, oxaliplatin treatment causes hyperexcitability and silencing of peripheral sensory neurons. We further show that oxaliplatin produces biphasic cold and mechanical hypersensitivities in mice. We expect that these findings will inform efforts to delineate the etiology of the short- and long-term sensory abnormalities reported by patients. Our findings support the hypothesis of A fiber dysfunction as a cause of dysesthesia and pain during and immediately after oxaliplatin transfusion.

## Author Contributions

**NS**, **NV**, **DAA**, and **SB**: Conceived the study and designed the experiments. **NS**, **NV**, **MRI**, and **CG**: Performed the experiments. **NS**, **MRI**, and **CG**: Analyzed the data. **NS**, **DA**, and **MRI**: Wrote the manuscript. **MRI**, **DAA**, and **SB**: Edited the manuscript. All authors have read and agreed to the published version of the manuscript.

## Disclosures

N.S. was supported by a BBSRC LIDo CASE PhD Studentship (awarded to D.A.A.), with support from Evotec. The work was supported by grants from the 10.13039/501100000265Medical Research Council (MR/L010747/1 to S.B., and MR/S003428/1 to D.A.A.), and from Versus Arthritis (21544 to D.A.A. and 21543 to S.B.). The authors declare no conflict of interest.
